# A Multicriteria Framework for Assessing the Sustainability of Viticulture and Winery Waste Valorization Routes under Cleaner Production and Circular Economy Principles

**DOI:** 10.1007/s00128-026-04353-5

**Published:** 2026-11-11

**Authors:** Luis F. O. Silva

**Affiliations:** Department of Civil and Environmental, Universidad de la Costa, Calle 58 #55-66, Barranquilla, 080002 Atlántico Colombia

**Keywords:** Circular economy, Cleaner production, Viticulture waste, Waste valorization, Sustainability assessment, Multicriteria framework

## Abstract

This review proposes a simple, broadly applicable multicriteria framework for classifying valorization routes for viticulture and winery waste under Cleaner Production and Circular Economy principles. The wine sector generates nutrient-rich by-products, yet most valorization studies evaluate isolated aspects and rarely integrate environmental benefits, contaminant risks, and circularity in a single comparison. This is important because a residue- management route may improve resource recovery while simultaneously increasing energy demand, transferring contaminants, or weakening material circularity. The framework combines six criteria, namely resource recovery efficiency, climate benefit, circularity potential, economic viability, environmental safety, and technological scalability, each scored semi-quantitatively using linguistic descriptors that are converted into numerical values, weighted, and aggregated into a composite score in which environmental safety acts as a constraint that cannot be offset by strong performance elsewhere. Using evidence from studies on grape by-products, we illustrate the framework and show how it flags routes that may transfer environmental burdens. The tool supports researchers, companies, and policy makers and can be adapted to other agro-industrial residues. Its principal purpose is screening and structured comparison rather than replacement of detailed life-cycle, economic, or toxicological assessments.

## Introduction

Grape cultivation is a major agro-industrial activity worldwide, and Brazil processes more than one million tonnes of grapes annually, with the southern region accounting for most national production (de Mello 2019; OIV 2018). Roughly one fifth of industrialized grapes can end up as by-products, mainly pomace, stems, and seeds, which may be discarded or poorly managed and thereby contribute to environmental contamination (Ferrari et al. 2019; Cortes et al. 2020). Turning these residues into useful products is therefore both an environmental opportunity and a potential source of value. Pressure to align industrial production with sustainability has moved the food and beverage sector toward the Circular Economy, which seeks to retain materials and resources in productive use while reducing waste and emissions (Murray et al. 2017; United Nations 2015). Food and food-processing residues are therefore relevant to this agenda, and composting of winery waste has been shown to provide environmental benefits when the resulting material substitutes mineral fertilizers (Principato et al. 2019; Cortes et al. 2020). Cleaner Production provides the complementary logic of preventing or minimizing impacts at the source. Many routes now compete to valorize viticulture and winery residues, including transformation of biomass into value-added products, nutrient-rich biofertilizers, carbon adsorbents for emerging contaminants, and recovery of polyphenols and oils (Laufenberg et al. 2003; Ferrari et al. 2019; Silva et al. 2021; Ky et al. 2014). Yet the literature evaluates these alternatives using disparate and often single-dimensional metrics, which makes them difficult to compare (Manara et al. 2015; Cortes et al. 2020). The aim of this Focused Review is to propose a simple multicriteria framework that places these routes on a common footing by integrating environmental benefit, contaminant risk, and circularity. The proposed approach is intentionally positioned between a purely qualitative discussion and a fully quantitative decision model. It does not attempt to generate a universal environmental score for each technology. Instead, it makes the criteria and assumptions visible, allows evidence from heterogeneous studies to be organized consistently, and provides a common structure that can subsequently be refined with measured indicators. This distinction is important because the evidence available for individual valorization routes is often uneven, particularly when laboratory-scale performance data are compared with industrial-scale or field-scale evidence.

## Limitations of Current Approaches

A first limitation is that most assessments focus on efficiency in a single dimension. Adsorbent studies emphasize removal capacity, agronomic studies emphasize nutrient availability, and life cycle studies emphasize greenhouse-gas balances, with little integration among these dimensions (Silva et al. 2021; Ferrari et al. 2019; Cortes et al. 2020). As a result, a route can appear excellent on one axis while drawbacks on other axes remain invisible. The problem is particularly relevant for residues because the same material can be viewed simultaneously as a feedstock, an agricultural input, an adsorbent precursor, or a source of energy. These functions imply different system boundaries and different definitions of success. A comparison based only on product yield or contaminant-removal efficiency can consequently favor a route whose upstream inputs, downstream emissions, or residual contaminants are not included in the assessment. The framework addresses this asymmetry by requiring every alternative to be described against the same six dimensions. A second limitation is the weak integration of environmental safety. Composting of grape residues can concentrate copper and chromium in the solid product to levels that may limit organic certification, and copper-based agricultural inputs can affect non-target organisms, as shown in studies of fruit flies and soil invertebrates (Ferrari et al. 2019; Rieder et al. 2024; Zamberlan et al. 2020; Naccarato et al. 2020). A route that improves nutrient supply but increases ecotoxicological risk may therefore transfer rather than eliminate an environmental burden (Rieder et al. 2023). A third limitation is the absence of standardized criteria for comparison, since functional units, system boundaries, and impact categories differ among studies (Thyberg and Tonjes 2017; Cortes et al. 2020). Without a shared scoring logic, decision makers cannot compare alternatives consistently, which motivates the framework proposed here (Murray et al. 2017). This limitation also affects interpretation across scales. A process that performs well under controlled laboratory conditions may require additional reagents, energy, transportation, or purification when scaled up. Conversely, a relatively simple route may have lower product value but provide broader material recovery with fewer technological barriers. Bringing these attributes into the same screening structure does not eliminate uncertainty, but it makes the source of uncertainty explicit and identifies where more quantitative information is most needed. A further consideration is that decision-analytic tools needed to overcome these limitations already exist but have rarely been applied to viticulture and winery residues. Coupling multicriteria decision analysis with life cycle assessment, and with risk assessment where contaminant hazards are relevant, offers a structured way to weigh criteria that cannot be reduced to a single monetary or environmental score (Linkov and Seager 2011; Seager and Linkov 2008). This coupling has been developed for emerging materials such as nanomaterials and for the management of contaminated media, where economic, environmental, safety, and risk attributes must be considered simultaneously (Seager and Linkov 2008; Linkov and Seager 2011). A recurring lesson from this body of work is that comparisons are sensitive to the weights assigned to each criterion and to uncertainty in the underlying inventory and fate models, making transparent weighting and explicit sensitivity analysis important (Prado et al. 2020; Mayo et al. 2014). The framework proposed here follows this tradition while remaining deliberately simple, so that it can be applied to grape and winery residues even when full quantitative datasets are not yet available.

## Proposed Assessment Framework

**Fig. 1:**
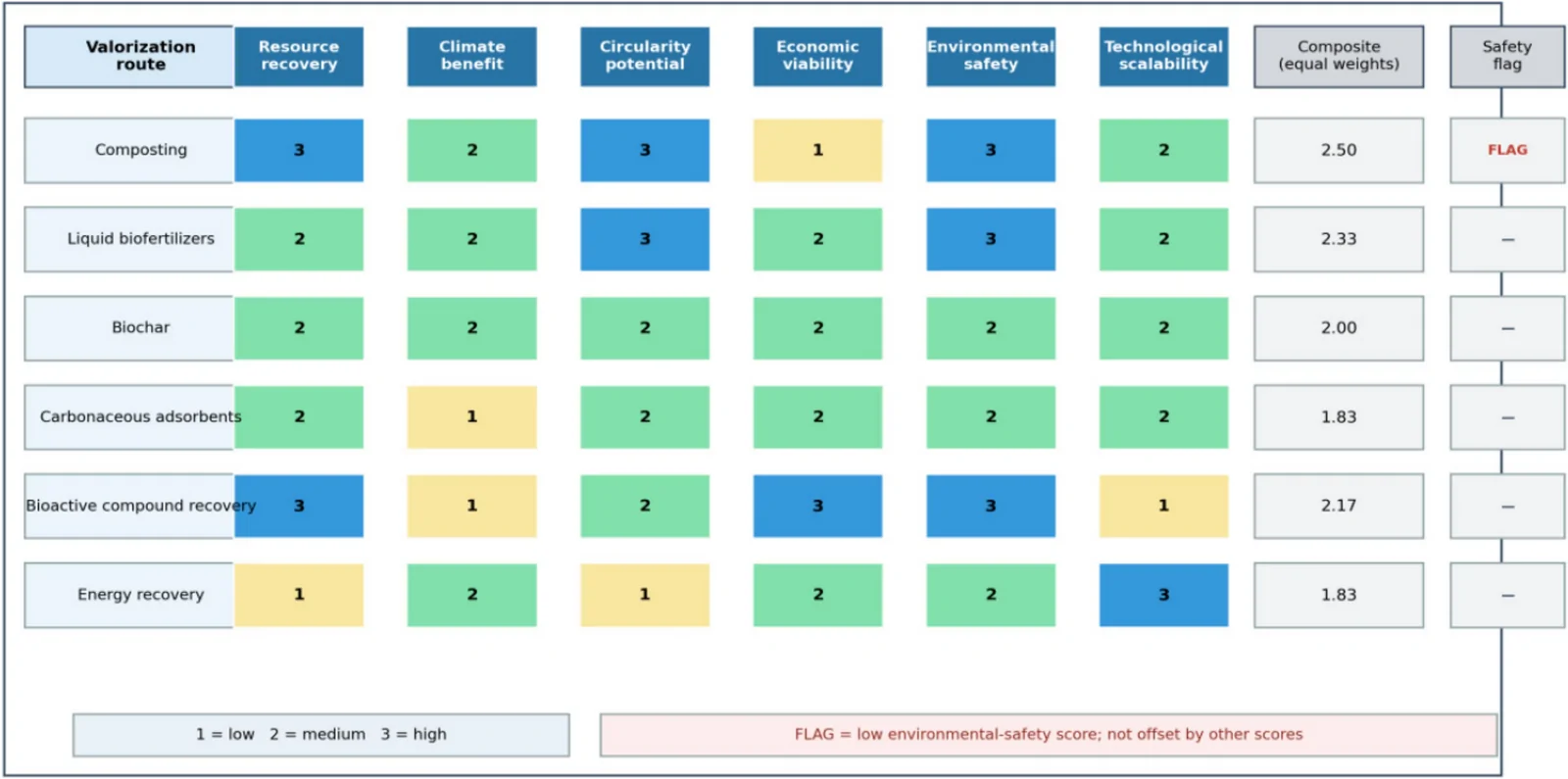
Illustrative application of the multicriteria framework to six valorization routes. Scores are semi-quantitative; the safety flag identifies a low environmental-safety score

We propose six criteria that can be scored semi-quantitatively, for example as low, medium, or high, so that each valorization route is assessed across all of them rather than on a single attribute. The conceptual scoring scheme is summarized in Fig. 1, and the criteria are defined in Table 1, drawing on life cycle and circularity reasoning (Cortes et al. 2020; Murray et al. 2017).

**Table 1:** The six criteria of the proposed framework, their definitions, and the evidence base that informs each one

Criterion	Definition	Evidence base
Resource recovery efficiency	Extent to which nutrients, organic carbon, bioactive compounds, or other valuable fractions are recovered for reuse	Ferrari et al. (2019); Ky et al. (2014); Silva et al. (2021)
Climate benefit	Net greenhouse gas balance, carbon storage, and emissions avoided by substituting mineral inputs	Cortes et al. (2020); Bong et al. (2017); Cobo et al. (2018)
Circularity potential	Capacity to close material loops and replace virgin raw materials rather than only divert waste	Murray et al. (2017); Oldfield et al. (2016); Mancini et al. (2019)
Economic viability	Processing cost, product value, and revenue potential	Duba and Fiori (2019); Yahya et al. (2015)
Environmental safety	Content of regulated contaminants and ecotoxicity toward non-target organisms	Ferrari et al. (2019); Rieder et al. (2024); Zamberlan et al. (2020)
Technological scalability	Readiness for industrial-scale operation and associated energy demand	Cortes et al. (2020); Manara et al. (2015); Silva et al. (2021)

The six criteria were selected to represent complementary dimensions of sustainability that are directly relevant to residue valorization. Importantly, they are not assumed to be independent. For example, technological scalability can influence economic viability and climate performance, while contaminant content can affect both environmental safety and the feasibility of returning a product to agricultural systems. Retaining them as separate criteria prevents one dimension from disappearing inside another and preserves the traceability of the assessment. The first criterion, resource recovery efficiency, captures the extent to which nutrients, organic carbon, bioactive compounds, or other valuable fractions are recovered for reuse, since grape residues are rich in both macroelements and high-value phenolics (Ferrari et al. 2019; Ky et al. 2014; Silva et al. 2021). The second criterion, climate benefit, reflects the net greenhouse gas balance, including carbon storage and the emissions avoided when compost or biofertiliser substitutes mineral inputs, while accounting for direct emissions during processing (Cortes et al. 2020; IPCC 2006; Bong et al. 2017; Cobo et al. 2018; Keng et al. 2020). The third criterion, circularity potential, measures how effectively a route closes material loops and replaces virgin raw materials rather than simply diverting waste from landfill (Murray et al. 2017; Oldfield et al. 2016; Mancini et al. 2019). The fourth criterion, economic viability, considers processing cost, product value, and revenue potential, which differ sharply between low-cost composting and higher-value but more expensive extraction routes (Duba and Fiori 2019; Yahya et al. 2015). The fifth criterion, environmental safety, weighs the content of regulated contaminants and the ecotoxicity of the product toward non-target organisms, an aspect that in vivo bioassays can quantify (Ferrari et al. 2019; Rieder et al. 2023, 2024; Zamberlan et al. 2020). The sixth criterion, technological scalability, assesses readiness for industrial operation and the associated energy demand, since some routes are already industrial while others remain at laboratory or pilot scale (Cortes et al. 2020; Manara et al. 2015; Silva et al. 2021). Taken together, the six criteria distinguish between recovery of material value and the broader conditions required for a route to remain environmentally defensible. This distinction is especially relevant for circular-economy applications because circularity alone does not guarantee low environmental risk. The framework consequently treats environmental safety as a criterion that must remain visible even when a route has strong resource-recovery or economic characteristics.

## Scoring, Weighting, and Aggregation

Each criterion is scored semi-quantitatively using three linguistic categories, namely low, medium, and high, which are assigned integer values of one, two, and three so that the assessment is explicit and reproducible (Ross and Sumner 2002). These concrete descriptors are anchored to the characteristics of each criterion and converted into numerical values for calculation. All criteria are oriented in the same direction, so that a higher value always denotes a more favourable outcome. For environmental safety, a high score therefore corresponds to a low content of regulated contaminants and low ecotoxicity toward non-target organisms (Rieder et al. 2023; Ferrari et al. 2019). Anchoring each category to concrete descriptors, including criterion-specific evidence where available, keeps the scoring comparable between assessors and studies. This explicit anchoring allows a deliberately qualitative screening tool to remain transparent and reproducible rather than arbitrary. The semi-quantitative nature of the scoring also makes the framework suitable for early-stage comparison. Assigning a value of 1, 2, or 3 is not intended to create false precision. Instead, it records whether the available evidence supports a relatively low, intermediate, or high performance level for a defined criterion. Where evidence is insufficient to justify a score, a future application should identify the information gap rather than infer a favourable value.

## Evidence Basis and Scoring Discipline

The quality of a multicriteria assessment depends not only on the number of criteria but also on the consistency with which evidence is translated into scores. For viticulture and winery residues, published evidence is heterogeneous: some studies report chemical composition, others report process performance, life-cycle indicators, ecotoxicity, or economic parameters. The framework therefore treats the score as a structured interpretation of available evidence rather than as a direct measurement. In an applied assessment, each score should be accompanied by a short evidence note identifying whether it is supported by measured data, a validated process study, a life-cycle assessment, toxicological testing, or expert judgement. This record would make the assessment auditable and would allow a later version of the analysis to replace provisional scores with quantitative indicators without changing the overall decision structure. A related safeguard is to distinguish evidence quality from technology popularity. A route should not receive a high score simply because it has been widely studied. Conversely, an emerging route should not automatically receive a low score when convincing pilot-scale or laboratory evidence supports its performance. The scoring should refer to the specific process and product under consideration. For example, a high-value extract may have strong economic potential, but this does not by itself establish economic viability if extraction, purification, transport, or quality-control requirements are substantial. The framework therefore encourages the assessor to document both the evidence supporting a score and the main uncertainty associated with it. The relative importance of the six criteria is expressed through weights that sum to one. In the absence of a specific decision context, we assign equal weights so that no single dimension dominates the comparison by default. Because comparisons are sensitive to the choice of weights, the framework is designed to accept context-specific weights elicited from stakeholders, for example giving greater weight to environmental safety in a protected watershed or to economic viability in a small enterprise (Linkov and Seager 2011; Prado et al. 2020). Making the weighting visible, rather than concealing it inside an aggregate score, is therefore a deliberate feature of the tool. An evidence register can further reduce a common problem in comparative sustainability studies: assigning a favourable value because an attribute is discussed frequently in the literature rather than because it has been demonstrated under conditions relevant to the intended application. Technological maturity should therefore be distinguished from the volume of published literature. Likewise, a process should not be considered environmentally safe solely because the original residue is natural or biodegradable. The relevant question is whether processing changes contaminant concentration, exposure pathways, emissions, or the fate of the recovered product. This distinction is particularly important for residues containing metals or agrochemical residues (Ali et al. 2019; Alengebawy et al. 2021; Naccarato et al. 2020). Equal weighting is therefore used only as a neutral illustrative baseline. In a real decision, stakeholders may assign different weights according to the intended use of the recovered material, local environmental constraints, market conditions, or regulatory requirements. Reporting the weights alongside the scores makes it possible to distinguish a change caused by evidence on an alternative from a change caused by the decision context itself. Scores and weights are combined through a weighted additive aggregation, so that the composite score of route j is the sum of its criterion scores multiplied by the corresponding weights: Sⱼ = Σ wᵢ xᵢⱼ (i = 1 to n) where Sⱼ is the composite score of route j, xᵢⱼ is the score of route j on criterion i, wᵢ is the weight of criterion i, the weights sum to one, and each xᵢⱼ takes the value one, two, or three. This additive form is transparent and easy to reproduce, but it is partially compensatory, meaning that a high score on one criterion can mask a low score on another. To prevent the tool from rewarding solutions that merely transfer environmental burdens, we treat environmental safety as a partially non-compensatory constraint, so that any route scoring low on this criterion is flagged regardless of its composite score (Seager and Linkov 2008). The composite score is therefore interpreted together with the safety flag rather than in isolation, keeping contaminant control central to the decision. This treatment differs from simply assigning environmental safety a larger numerical weight. A weighted average remains compensatory by construction, whereas the safety flag identifies a condition that may be unacceptable even when other attributes are strong. The approach therefore preserves the usefulness of a simple composite while preventing the composite from becoming the sole basis for interpreting environmental acceptability.

## Treatment of Uncertainty and Decision Context

**Fig. 2:**
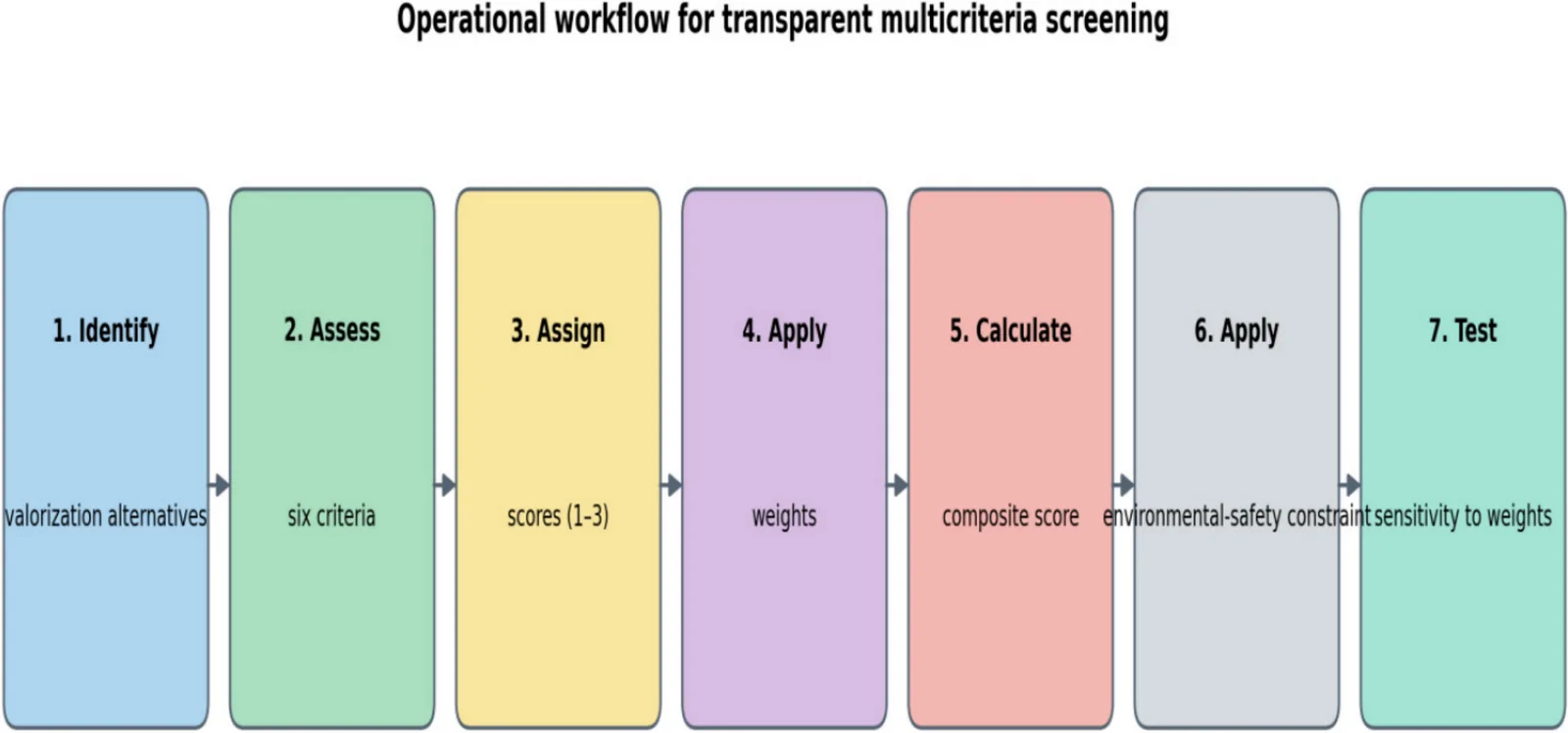
Operational workflow for applying the proposed multicriteria framework

Uncertainty enters the framework at several levels. The first concerns the evidence itself, including variability in feedstock composition and differences between laboratory, pilot, and industrial conditions. The second concerns the decision context, because the same product may have different value depending on local markets, fertilizer demand, energy prices, or regulatory requirements. The third concerns preferences, which are represented by the weights. These forms of uncertainty should not be collapsed into a single numerical correction. Instead, they can be examined separately through scenario-based sensitivity analysis. One scenario can emphasize environmental safety, another resource recovery, and another a small-enterprise context in which capital expenditure and technological complexity are particularly important. If a route remains acceptable across these scenarios, confidence in the screening conclusion increases; if its position changes substantially, the result should be interpreted as context dependent. The framework also permits a distinction between screening thresholds and final decision thresholds. A screening threshold identifies alternatives that require additional investigation, whereas a regulatory or operational threshold determines whether implementation is permitted or appropriate in a specific setting. The present framework addresses the former. This distinction is important for environmental contamination because regulatory limits depend on the intended use, exposure pathway, jurisdiction, and environmental compartment. A generic multicriteria score should therefore not be interpreted as evidence of regulatory compliance. Instead, a low environmental-safety score should trigger targeted chemical and toxicological characterization before a route is advanced. Because both scores and weights carry uncertainty, we recommend that any application of the framework be accompanied by a simple sensitivity analysis in which the weights are varied within plausible ranges to test whether the comparison of routes is robust (Prado et al. 2020; Mayo et al. 2014). Figure 2 summarizes the operational workflow, whereas Fig. 1 presents the illustrative scoring matrix for the six routes discussed below. A matrix representation is used in preference to a radar or spider diagram because the area enclosed by a radar plot depends on the arbitrary ordering of its axes and can convey a misleading impression of overall performance, whereas a matrix reports each score independently. This choice keeps the visual comparison faithful to the underlying scores. The workflow in Fig. 2 makes this sequence explicit: alternatives are defined, the six criteria are scored, weights are applied, the composite is calculated, and the environmental-safety constraint and sensitivity of the comparison are examined. This sequence also separates assessment from interpretation. The numerical composite summarizes the chosen assumptions, whereas the safety flag and sensitivity analysis indicate whether those assumptions conceal an important trade-off.

## Application of the Framework

Table 2 applies the framework qualitatively to six representative routes. Composting scores well on resource recovery, circularity, and scalability because it is industrially mature and can substitute for mineral fertilizer, but diesel use and direct gaseous emissions are environmental hotspots, and the product can concentrate copper and chromium (Cortes et al. 2020; Ferrari et al. 2019; Bong et al. 2017; Saer et al. 2013; Andersen et al. 2010).

**Table 2:** Qualitative comparison of six valorization routes for viticulture and winery waste, with their main advantages and limitations

Valorization route	Main advantages	Main limitations
Composting	Recovers organic matter and nutrients, substitutes mineral fertiliser, industrially mature (Cortes et al. 2020; Bong et al. 2017)	Diesel and direct gaseous emissions are hotspots; product can concentrate copper and chromium, limiting organic certification (Cortes et al. 2020; Ferrari et al. 2019)
Liquid biofertilizers	Low contaminant load, biologically safe in non-target bioassays, supports nutrient supply (Rieder et al. 2023; Mahanty et al. 2017)	Lower nutrient concentration; depends on consistent feedstock (Ferrari et al. 2019)
Biochar	Carbon storage potential, soil amendment, contaminant adsorption (Silva et al. 2021; Li et al. 2019)	Energy demand of pyrolysis; performance depends on feedstock (Silva et al. 2021)
Carbonaceous adsorbents	High removal of emerging contaminants, valorises low-value residue (Silva et al. 2021; Sophia and Lima 2018)	Chemical activation reagents and acid washing; limited reuse (Angin 2014; Silva et al. 2021)
Bioactive compound recovery	High-value polyphenols and oils with market revenue (Ky et al. 2014; Gullon et al. 2017)	Extraction cost and solvent or supercritical-CO₂ scale-up challenges (Duba and Fiori 2019; Kopsahelis et al. 2018)
Energy recovery	Reduces residue volume and recovers calorific value (Manara et al. 2015; Laufenberg et al. 2003)	Lower circularity since nutrients are not returned to soil; emissions require control (Cortes et al. 2020)

## Interpretation of the Illustrative Comparison

The values in Table 2 and Fig. 1 should be interpreted as an illustration of how the framework operates rather than as a universal ranking of technologies. The purpose is to demonstrate the information that becomes visible when the same criteria are applied to different routes. Composting, for example, combines a mature technology with substantial nutrient and organic-matter recovery, but environmentalsafety considerations may become important when metals are concentrated in the final product. Liquid biofertilizers can have a favourable safety profile while remaining dependent on feedstock consistency and nutrient concentration. Biochar and carbonaceous adsorbents provide a pathway for recovering carbon-rich fractions and treating contaminated streams, but their performance depends strongly on preparation conditions, energy inputs, and management of spent materials. Bioactive-compound recovery can create high-value products, yet extraction intensity and market requirements may constrain its overall viability. Energy recovery can reduce residue volume and recover calorific value, but it generally returns less material to productive cycles than routes that preserve nutrients or carbon. These contrasts demonstrate why a single indicator can be misleading. A route with high resource recovery may not have the lowest climate burden, and a route with high economic potential may not provide the safest environmental endpoint. The composite score is useful because it summarizes the selected dimensions, but the safety flag remains necessary because environmental acceptability is not necessarily compensatory. In a real application, the assessor should therefore report both the individual criterion scores and the composite result. Reporting only the aggregate would obscure which attribute controls the conclusion. The illustrative comparison also shows how the framework can guide evidence collection. If two routes have similar composite scores but differ strongly in environmental safety, the next research priority should concern contaminant characterization or ecotoxicity rather than a further refinement of a minor economic difference. If two routes differ mainly in technological scalability, pilot-scale experiments may be more informative than additional laboratory optimization. The framework thus connects comparative assessment with research prioritization and provides a rational basis for deciding where additional evidence is likely to reduce decision uncertainty most effectively. In practical use, the framework should be applied iteratively. An initial screening can identify routes that merit detailed investigation; subsequent chemical characterization, ecotoxicological testing, life-cycle assessment, and economic analysis can replace provisional scores as quantitative evidence becomes available. This staged approach is useful when several alternatives are technically feasible but resources for full assessment are limited, and it makes uncertainty an explicit research priority rather than hiding it within a single aggregate value. Liquid biofertilizers obtained from grape extracts score highly on environmental safety because they carry low contaminant loads and showed no toxicity in non-target fruit-fly assays, although their nutrient concentration is lower and depends on consistent feedstock (Rieder et al. 2023; Ferrari et al. 2019; Mahanty et al. 2017). Biochar and carbonaceous adsorbents score well for contaminant removal and for valorizing otherwise low-value composting residues, yet they require energy for pyrolysis or chemical activation and acid washing, which affects their climate and environmental-safety performance (Silva et al. 2021; Li et al. 2019; Angin 2014; Sophia and Lima 2018). These examples also show why the same route may perform differently when the functional unit changes. A composting system evaluated per tonne of residue may look different from one evaluated per kilogram of recovered nutrient or per unit of avoided mineral fertilizer. Similarly, an adsorbent route can have strong contaminant-removal performance per gram of material while requiring additional energy or chemicals per unit of treated wastewater. The framework does not remove these differences; it makes them visible by requiring the assessor to state what is being recovered, what environmental benefit is expected, and what additional burden is created. For a full application, the criterion descriptors should therefore be accompanied by a clearly stated functional unit and system boundary. A further distinction concerns the destination of the recovered product. Products intended for direct agricultural application have a different safety profile from products used as industrial raw materials, and both differ from energy recovery in which the principal objective is fuel substitution. This is one reason environmental safety is retained as a separate criterion rather than inferred from circularity. The destination determines the potential exposure pathway and therefore the type of evidence required. For soil amendments, contaminant concentrations and non-target biological effects are central; for industrial adsorbents, the fate of captured contaminants and management of spent material become more important; and for energy recovery, emission controls and ash management are critical considerations. Recovery of bioactive compounds can provide high economic value because polyphenols and grape-seed oils can command premium markets, but extraction and supercritical-CO₂ scale-up raise cost and energy considerations (Ky et al. 2014; Gullon et al. 2017; Kopsahelis et al. 2018; Duba and Fiori 2019). Energy recovery reduces residue volume and recovers calorific value, yet it tends to score lower on circularity because it does not return nutrients to the soil, and its emissions require control (Manara et al. 2015; Laufenberg et al. 2003; Cortes et al. 2020). In Fig. 1, composting has a high equal-weight composite but is flagged by the environmental-safety constraint, illustrating how the tool separates overall performance from a specific risk of burden transfer.

## Strengths and Limitations of the Screening Approach

The principal strength of the framework is transparency. A decision maker can see which criteria were considered, which weights were applied, and why an alternative received a particular qualitative score. This is preferable to presenting a single sustainability index whose internal assumptions are difficult to inspect. The framework is also modular: criteria can be refined when better data become available, and additional criteria can be introduced for residues with specific hazards. This flexibility is relevant to agro-industrial wastes because composition and processing conditions vary among regions and production systems. Several limitations should nevertheless be recognized. First, the three-level scoring system cannot represent small but potentially meaningful differences between alternatives. Second, equal weighting is illustrative and does not represent the preferences of a particular stakeholder group. Third, the framework depends on the quality and comparability of published evidence, which can be limited for emerging technologies. Fourth, the environmental-safety constraint is a screening safeguard rather than a substitute for formal risk assessment. Finally, the framework does not calculate monetary costs, greenhouse-gas emissions, or toxicity indicators itself; it provides a structure within which such quantitative indicators can subsequently be incorporated. These limitations are deliberate consequences of keeping the tool simple enough for early-stage comparison. Future applications can address these limitations in stages. The first stage can retain the present 1–3 scoring scheme and document the evidence behind each score. The second can replace selected qualitative scores with quantitative indicators, such as measured contaminant concentrations, life-cycle greenhouse-gas emissions, energy demand, or product yield. The third can apply formal multicriteria decision-analysis methods with stakeholder-derived weights and uncertainty distributions. In this progression, the present framework functions as a common conceptual scaffold rather than a final analytical endpoint.

## Implications for the Circular Economy

**Fig. 3:**
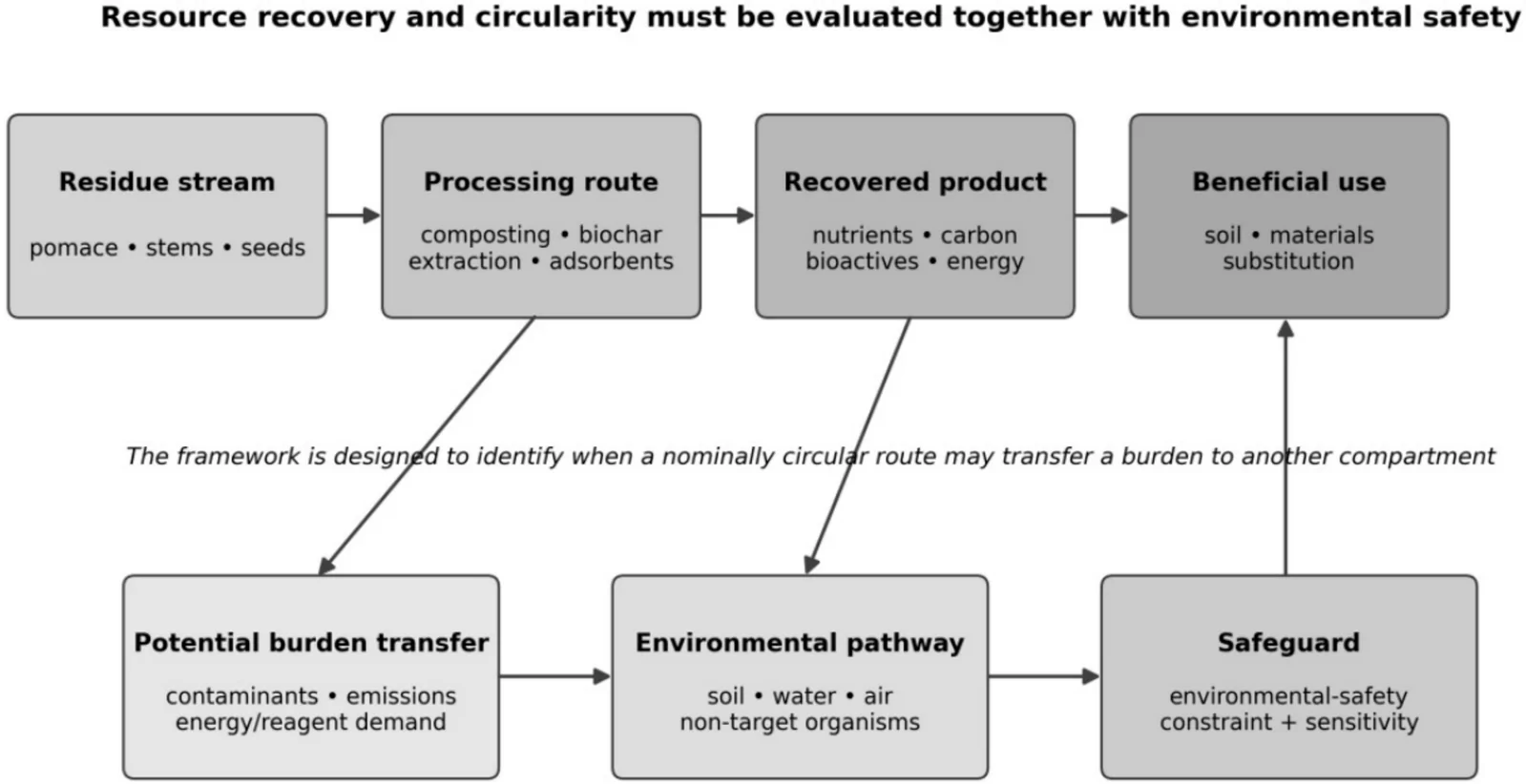
Conceptual relationship between resource recovery, valorization, and potential environmental burden transfer. The environmental-safety constraint provides a safeguard before recovered products are returned to use

The main value of the framework is to discourage solutions that merely shift impacts from one environmental compartment to another, for example by reducing landfill disposal while increasing ecotoxicological risk in soil or water (Murray et al. 2017; Cobo et al. 2018; Thyberg and Tonjes 2017). By requiring every route to be assessed against an environmental-safety criterion, the tool keeps Cleaner Production goals and contaminant control within the same decision framework (Rieder et al. 2024). The framework also helps distinguish between two forms of circularity that can otherwise be conflated. The first is material recirculation, in which nutrients, carbon, or bioactive compounds are returned to productive use. The second is environmental performance, which concerns whether that recirculation occurs without creating a new contaminant pathway or disproportionate processing burden. These dimensions can move in different directions. A residue may be efficiently converted into a useful product while concentrating a metal or requiring an energy-intensive treatment step. Conversely, a low-complexity route may provide modest economic value but retain more of the original material in a useful cycle. Figure 3 summarizes this distinction conceptually and shows why the environmental-safety constraint is positioned between the valorization pathway and its environmental consequences.

## Transferability to Other Agro-Industrial Residues

The framework can be adapted to olive-mill residues, sugarcane bagasse, coffee-processing residues, fruit-processing wastes, and other organic industrial streams. The six core criteria remain relevant because each route must balance recovery of resources against environmental safety, climate effects, economic feasibility, and scalability. However, the descriptors used to assign scores should be modified to reflect the characteristic contaminants and products of each residue. A residue associated with pesticide use may require stronger emphasis on pesticide residues, whereas a mineral-rich residue may require greater attention to metals or salinity. Transferability therefore concerns the decision structure, not the assumption that identical thresholds apply to every material. The framework is also compatible with more formal life-cycle and risk-assessment approaches. Once quantitative inventories become available, the six criteria can be linked to measured indicators rather than qualitative scores. For example, climate benefit could be expressed through greenhouse-gas emissions per functional unit, while environmental safety could incorporate measured contaminant concentrations, exposure estimates, and ecotoxicological endpoints. This progression would preserve the conceptual advantages of the framework while increasing quantitative resolution. The present version is therefore best viewed as a transparent bridge between heterogeneous evidence and more data-intensive assessment methods.

## Recommended Implementation Sequence

For practical use, the framework can be implemented in five stages. First, the assessor defines the residue stream, the intended product, the geographical and technological boundary, and the functional unit. Second, all candidate routes are described using the same boundary and the same six criteria. Third, evidence is collected for each criterion and recorded together with its source, scale of observation, and principal uncertainty. Fourth, the 1–3 scores and weights are assigned, the composite is calculated, and the environmentalsafety constraint is applied. Fifth, the most decision-relevant uncertainties are tested through sensitivity analysis and, where necessary, targeted laboratory or field measurements. This sequence prevents a favourable technology from being selected simply because its advantages were measured more thoroughly than those of competing routes. The implementation sequence also creates a clear interface between screening and experimental research. If environmental safety controls the decision, analytical effort can be directed first toward contaminant concentrations, chemical speciation, mobility, and biological effects. If scalability is limiting, pilot-scale energy demand, throughput, reagent consumption, and product consistency become priorities. If economic viability is uncertain, process yield, product-market assumptions, capital requirements, and operating costs should be refined. In this way, the framework does not merely rank alternatives; it can help identify the next most informative measurement or experiment. This research-planning function is particularly valuable for emerging valorization technologies for which evidence is fragmented and resources for comprehensive assessment are limited. The environmental-safety constraint provides a safeguard before recovered products are returned to use. This perspective is consistent with the preventive logic of Cleaner Production. A circular route is not environmentally preferable merely because it diverts material from disposal; its downstream use must also avoid creating a new exposure pathway or concentrating contaminants in a product that is subsequently released to the environment. The framework therefore emphasizes the complete valorization chain, from residue generation and processing to product use and management of remaining fractions. A practical implication is to embed simple non-target bioassays, such as Drosophila melanogaster development and behavior tests, into the screening of new biofertilizers and amendments, since such assays can detect biological effects at relatively low cost (Rieder et al. 2023, 2024; Zamberlan et al. 2020). Coupled with attention to heavy-metal and rare-earth-element contamination of agricultural inputs, this approach can inform future research on safer circular valorization routes (Ali et al. 2019; Naccarato et al. 2020; Alengebawy et al. 2021).

## Implications for Environmental Monitoring

Environmental monitoring should follow the pathway created by the valorization process rather than focus exclusively on the original residue. If a treatment concentrates metals in a solid fraction, the subsequent use of that fraction determines the relevant exposure route. If chemical activation is used to produce an adsorbent, the fate of residual reagents and the contaminants captured during use becomes part of the environmental-safety assessment. If nutrients are recovered for agricultural application, soil accumulation and effects on non-target organisms become relevant. This pathway-oriented perspective links the framework to Cleaner Production because it directs attention toward prevention of new releases at the point where a residue is transformed into a product. The same logic supports a staged monitoring strategy. Initial screening can use bulk chemical characterization and a small set of ecotoxicological endpoints; routes that remain promising can then receive more detailed analysis of contaminant speciation, mobility, transformation, and long-term exposure. Such sequencing can be more informative than applying the same analytical intensity to every route. It also connects the multicriteria framework with the practical allocation of research resources, because the criteria can identify which environmental pathway is most likely to control the decision.

## Conclusions

This Focused Review offers a simple multicriteria framework to support decisions on the sustainable management of viticulture and winery waste. By scoring resource recovery, climate benefit, circularity, economic viability, environmental safety, and technological scalability together, and by combining them through transparent weighted aggregation in which environmental safety cannot be traded away, the framework reveals trade-offs that singledimension assessments can miss and flags routes that may transfer environmental burdens. The qualitative scoring keeps the tool accessible to researchers, companies, and policy makers, and its structure is general enough to be adapted to other agro-industrial residues. Future work can refine the criteria into quantitative indicators, formalize the weighting through participatory elicitation, and test the framework against full life-cycle and ecotoxicity datasets. The framework is particularly useful at the stage when several technically plausible routes must be narrowed to a smaller set for detailed investigation, because it makes trade-offs explicit without implying false precision.

## Reporting and Reproducibility Considerations

To facilitate reuse of the framework, an applied study should report the residue characteristics, intended destination of the recovered product, system boundary, functional unit, evidence source for each criterion, assigned score, criterion weight, composite score, and result of the environmental-safety constraint. This information would allow another assessor to reproduce the screening exercise and to identify which assumptions are responsible for differences between applications. Where a score is based primarily on expert judgement, that fact should be stated explicitly and, where possible, accompanied by a brief rationale. The framework is deliberately compatible with progressively more quantitative reporting. For example, the qualitative climate-benefit score can later be replaced by a life-cycle greenhouse-gas indicator, while the environmental-safety score can be informed by measured concentrations, bioavailability, exposure estimates, or non-target toxicity. Similarly, economic viability can incorporate capital expenditure, operating cost, product price, and avoided disposal costs when such information becomes available. Keeping the conceptual criteria stable while improving the evidence behind individual scores provides continuity between an early screening assessment and a subsequent detailed study. This reporting discipline is also important for avoiding false precision: a numerical composite should remain traceable to the evidence and assumptions from which it was derived.
